# A Highly Specific Monoclonal Antibody for Botulinum Neurotoxin Type A-Cleaved SNAP25

**DOI:** 10.3390/toxins7072354

**Published:** 2015-06-24

**Authors:** Catherine Rhéaume, Brian B. Cai, Joanne Wang, Ester Fernández-Salas, K. Roger Aoki, Joseph Francis, Ron S. Broide

**Affiliations:** 1 Department of Biological Sciences, Allergan, Irvine, CA 92612, USA; E-Mails: rheaume_catherine@allergan.com (C.R.); cai_brian@allergan.com (B.C.); wang_joanne@allergan.com (J.W.); aoki.roger.7@gmail.com (K.R.A.); joe-francis@cox.net (J.F.); 2 Department of Pathology, School of Medicine, University of Michigan, Ann Arbor, MI 48109, USA; E-Mail: esterf@med.umich.edu

**Keywords:** botulinum neurotoxin, SNAP25, neuromuscular junction, motor nerve, autonomic nerve, recombinant antibody

## Abstract

Botulinum neurotoxin type-A (BoNT/A), as onabotulinumtoxinA, is approved globally for 11 major therapeutic and cosmetic indications. While the mechanism of action for BoNT/A at the presynaptic nerve terminal has been established, questions remain regarding intracellular trafficking patterns and overall fate of the toxin. Resolving these questions partly depends on the ability to detect BoNT/A’s location, distribution, and movement within a cell. Due to BoNT/A’s high potency and extremely low concentrations within neurons, an alternative approach has been employed. This involves utilizing specific antibodies against the BoNT/A-cleaved SNAP25 substrate (SNAP25_197_) to track the enzymatic activity of toxin within cells. Using our highly specific mouse monoclonal antibody (mAb) against SNAP25_197_, we generated human and murine recombinant versions (rMAb) using specific backbone immunoglobulins. In this study, we validated the specificity of our anti-SNAP25_197_ rMAbs in several different assays and performed side-by-side comparisons to commercially-available and in-house antibodies against SNAP25. Our rMAbs were highly specific for SNAP25_197_ in all assays and on several different BoNT/A-treated tissues, showing no cross-reactivity with full-length SNAP25. This was not the case with other reportedly SNAP25_197_-selective antibodies, which were selective in some, but not all assays. The rMAbs described herein represent effective new tools for detecting BoNT/A activity within cells.

## 1. Introduction

Botulinum neurotoxin type A (BoNT/A) causes transient muscle paralysis through presynaptic blockade of acetylcholine release at the neuromuscular junction (NMJ). The therapeutic utility of BoNT/A has grown considerably over the past several decades and Allergan’s product, onabotulinumtoxinA, is now approved globally for 11 (10 US) major therapeutic and cosmetic indications [1,2]. These indications include treatment for disorders of skeletal muscle (e.g., cervical dystonia, upper and lower limb spasticity) [3,4], smooth muscle (e.g., idiopathic and neurogenic detrusor overactivity) [5,6], autonomic nerves (e.g., axillary hyperhidrosis) [7] and nociceptive pain mechanisms (e.g., chronic migraine) [8,9].

While the general mechanism of action (MoA) for BoNT/A at the presynaptic nerve terminal has been well established [10,11], there are still many unanswered questions regarding the intracellular trafficking patterns and general “life-cycle” of the toxin. Resolving these questions partly depends on the ability to precisely detect the toxin’s location, distribution, and movement within a cell. Direct detection of BoNT/A using antibodies has been difficult due to its high potency and therefore, extremely low concentrations within neurons [12,13]. An alternative approach for detecting the presence of BoNT/A has been to track its enzymatic activity via immuno-staining for the cleaved SNAP25 product.

Following internalization and translocation of the light chain into the cytosol, BoNT/A cleaves its substrate SNAP25 at amino acid 197 (SNAP25_197_), removing the last nine amino acids of the protein [14,15]. The resulting new terminal epitope that is generated is difficult to specifically target with an antibody without also recognizing the intact SNAP25 protein [16]. Additionally, the specificity of any given SNAP25_197_ antibody may be assay, tissue or species-dependent. This would suggest that an antibody to SNAP25_197_ should ideally be tested under multiple conditions and tissue types to determine its specificity for BoNT/A-cleaved SNAP25 in all applications.

A number of recently published studies have employed either a proprietary rabbit polyclonal antibody [17,18,19,20,21] or a commercially-available monoclonal antibody [22,23] to track SNAP25_197_ presence in the rodent central nervous system and bladder, respectively. The SNAP25_197_-positive staining observed in these studies was purported as being indicative for the presence of active BoNT/A. However, various inconsistencies between these studies and the lack of specific control experiments suggest that the observed SNAP25_197_ immuno-reactive (IR) signal may misrepresent the neuronal trafficking patterns of the active toxin.

In an effort to address BoNT/A mechanistic questions, we have generated highly specific mouse monoclonal antibodies (mAb) against SNAP25_197_ using a twelve residue peptide antigen containing amino acids 186-197 from the terminal carboxylated region of the BoNT/A-cleaved SNAP25 protein. One of these mAbs (2E2A6) is used in Allergan’s cell-based potency assay for BOTOX^®^ (Allergan, Irvine, CA, USA) [24,25]. The variable regions of a second mAb (3C1A5), selected for its superior performance in immunohistochemical (IHC) assays on muscle tissue [26], were sequenced and recombinantly engineered into immunoglobulin backbones from either human (IgG1) or murine (IgG2A) origin. This was done in order to reduce background and cross-staining and to allow for species-specific co-localization studies. In the present study, we validated the specificity of our recombinant monoclonal antibodies (rMAb) in several different assays and performed a side-by-side comparison with various commercially-available and in-house produced antibodies directed against SNAP25.

## 2. Results

### 2.1. Western Blot Analysis

**Figure 1:**
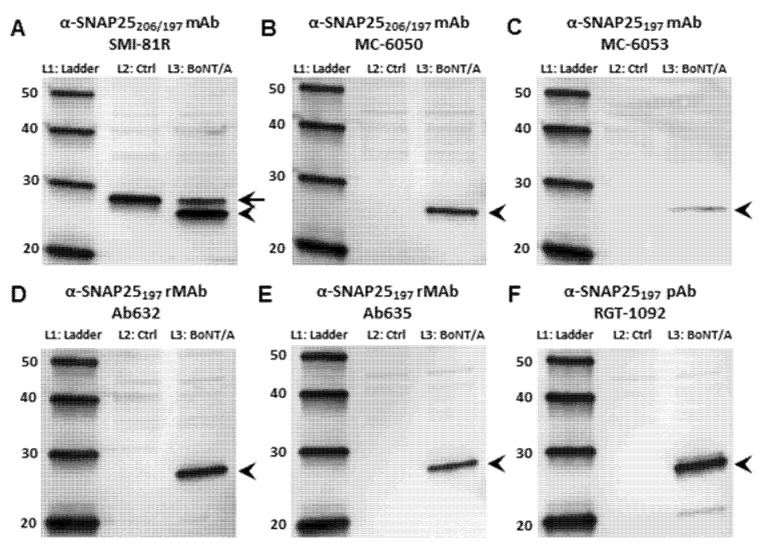
Western Blot analysis comparing the specificity of antibodies against SNAP25 using rat embryonic cortical neuronal cell lysates treated with (L_3_) or without (L_2_) BoNT/A. (**A**) Blot probed with a commercially available anti-SNAP25 mAb (SMI-81R) that recognizes both the full-length (206) and cleaved (197) forms of SNAP25. In lane 2, only SNAP25_206_ is detected, whereas in lane 3, both SNAP25_206_ (arrow) and SNAP25_197_ (arrowhead) are detected; (**B**) Blot probed with a commercially available anti-SNAP25 mAb (MC-6050) that reportedly recognizes both SNAP25_206_ and SNAP25_197_. Only SNAP25_197_ appears as a single band in lane 3 (arrowhead); (**C**) Blot probed with a commercially available anti-SNAP25 mAb (MC-6053) that is reportedly specific for SNAP25_197_. This antibody recognizes a thin, faint SNAP25_197_ band in lane 3; (**D**) Blot probed with Ab632 anti-SNAP25_197_ rMAb. In lane 2, no band is detected, whereas in lane 3, a single band for SNAP25_197_ is detected (arrowhead); (**E**) Blot probed with Ab635 anti-SNAP25_197_ rMAb. In lane 2, no band is detected, whereas in lane 3, a single band for SNAP25_197_ is detected (arrowhead); (**F**) Blot probed with RGT-1092 anti-SNAP25_197_ pAb. This antibody primarily recognizes SNAP25_197_ in lane 3 (arrowhead), although two faint bands are visible just above and below the SNAP25_197_ band. Lane 1, protein ladder; Lane 2, untreated cortical cell lysate; Lane 3, BoNT/A-treated (3 nM) cortical cell lysate.

The primary antibodies used in the current study are listed in Table 1. The antibody comparison was first performed using Western blot (WB) analysis. We compared our recombinant anti-SNAP25_197_ monoclonal antibodies against both commercially available antibodies and our in-house rabbit polyclonal antibody (pAb) [27] in their ability to recognize the full-length (206) or BoNT/A-cleaved (197) forms of SNAP25 from rat embryonic cortical cell lysates treated with or without BoNT/A (Figure 1). A commercially available and widely-used monoclonal antibody (SMI-81R) directed against all forms of the SNAP25 protein recognized both SNAP25_206_ and SNAP25_197_ (Figure 1A). In contrast, a second commercially available monoclonal antibody (MC-6050) described [28] as recognizing both forms of SNAP25 was surprisingly specific for SNAP25_197_ in lysates from toxin-treated cells (Figure 1B). Furthermore, another antibody from the same company (MC-6053), described [29] as recognizing only BoNT/A-cleaved SNAP25 revealed a thin, faint band exclusively in the toxin-treated lane (Figure 1C).

Our human (Ab632) and murine (Ab635) rMAbs directed against BoNT/A-cleaved SNAP25 were very specific for SNAP25_197_; only a single band was detected in toxin-treated lysates, while no bands were detected in the untreated, control lanes (Figure 1D, E). Similarly, our in-house rabbit pAb (RGT-1092) against SNAP25_197_ only detected a band in the BoNT/A-treated sample, while no bands were detected in the control lane (Figure 1F). We did note however, that the pAb recognized two additional faint bands, one just above the SNAP25_197_ band and another at ~20 kDa present only in toxin-treated samples, which could not be readily explained. Nevertheless, the upper band is not likely to be intact SNAP25, as no band was observed in the untreated lane. Similar WB results were obtained using lysates from BoNT/A-treated and untreated SiMa cell cultures (Figure S1), except that no band was observed for MC-6053 in either control or toxin-treated lanes (Figure S1C). Control blots probed for GAPDH showed equal loading of all samples for both cortical and SiMa cell lysate experiments (Figure S2).

A separate WB analysis was performed to examine the epitope specificity of our rMAbs using BoNT/C- and BoNT/E-treated SiMa cell lysates compared to the BoNT/A-treated lysate. It is well established that BoNT/C cleaves SNAP25 at amino acid (aa) residue 198, while BoNT/E cleaves SNAP25 at aa residue 180 [11]. While the SMI-81R mAb recognized both full length and BoNT-cleaved forms of SNAP25 (Figure S2C), our human rMAb only detected a single band in the BoNT/A-treated lysate sample, as expected (Figure S2D).

**Table 1:** List of anti-SNAP25 antibodies used in the current study.

Antibody	Specificity	Vendor	Species/Type	IgG-isotype	SNAP25_197_ antigen
SMI-81R	SNAP25_206/197_	Covance, Princeton, NJ	Murine/mAb	IgG1	Uncleaved SNAP25
MC-6050	SNAP25_206/197_	R&D Abs, LV, NV	Murine/mAb	n/a	15-mer, C_OOH_-term
MC-6053	SNAP25_197_	R&D Abs, LV, NV	Murine/mAb	n/a	15-mer, C_OOH_-term
Ab632	SNAP25_197_	Allergan	rHuman/rMAb	IgG1	12-mer, C_OOH_-term
Ab635	SNAP25_197_	Allergan	rMurine/rMAb	IgG2A	12-mer, C_OOH_-term
RGT-1092	SNAP25_197_	Allergan	Rabbit/pAb	IgG	7-mer, C_OOH_-term

n/a = not available; mAb = mouse monoclonal antibody; rMAb = recombinant monoclonal antibody; pAb = rabbit polyclonal antibody.

### 2.2. Immunohistochemistry

**Figure 2:**
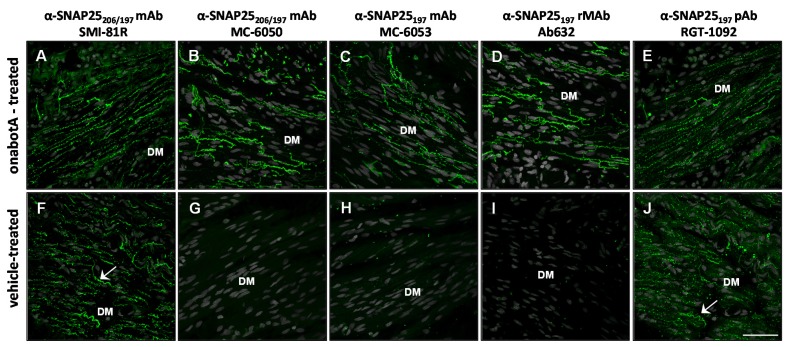
Immunohistochemical analysis comparing the specificity of antibodies against SNAP25 in sections of rat bladder following treatment with either onabotulinumtoxinA (10 U/kg) or vehicle. (**A**–**E**) Confocal images of bladder detrusor muscle (DM) injected with onabotulinumtoxinA and probed with (**A**) commercial mAb (SMI-81R) against full-length (206) and cleaved (197) SNAP25; (**B**) a second commercial mAb (MC-6050) against SNAP25_197_ & SNAP25_206_; (**C**) commercial mAb (MC-6053) against SNAP25_197_; (**D**) Ab632-rMAb against SNAP25_197_ and (**E**) RGT-1092 pAb against SNAP25_197_; (**F**–**J**) Confocal images of control rat bladder injected with vehicle and probed with the same five antibodies. SNAP25-IR signal is in green. Arrows ((**F**) and (**J**)) point to IR-signal within nerve fibers from vehicle treated rat bladder. DM, detrusor muscle; Scale bar = 50 µm.

The specificity of the antibodies was then tested using immunohistochemistry (IHC) in rat bladder and glabrous skin that had been treated with either onabotulinumtoxinA or saline. Throughout these IHC studies, adjacent sections processed without primary antibodies showed only background staining (Figure S3E,F). In rat bladder, the SMI-81R antibody directed against SNAP25 showed IR-signal in nerve fibers throughout the detrusor muscle. The SNAP25-IR pattern was identical in both onabotulinumtoxinA and saline-treated bladders, as expected (Figure 2A,F). The MC-6050 monoclonal antibody, which is reported to recognize both intact and BoNT/A-cleaved forms of SNAP25, demonstrated IR-signal in nerve fibers from the detrusor muscle of toxin-treated, but not saline-treated bladder (Figure 2B,G), comparable to the results from the Western blot analysis. Likewise, the MC-6053 monoclonal antibody demonstrated IR-signal only in toxin-treated, but not saline-treated bladder (Figure 2C,H). In contrast with the specificity detected in the WB analysis, RGT-1092 pAb generated against SNAP25_197_ demonstrated IR-signal in both toxin-treated and saline-treated bladders (Figure 2E,J). Most importantly, Ab632-rMAb showed clear IR-signal in nerve fibers from the detrusor muscle of toxin-treated bladders (Figure 2D), while no signal was detected in saline-treated control bladders (Figure 2I). Moreover, in separate studies, Ab635-rMAb showed similar specificity as Ab632-rMAb to SNAP25_197_ in rat bladder (Figure S3A,C).

**Figure 3:**
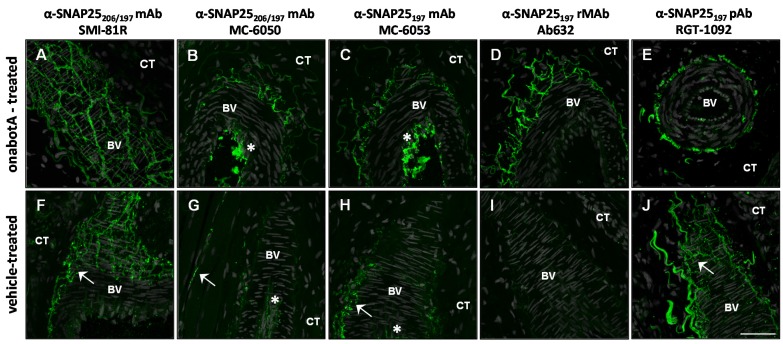
Immunohistochemical analysis comparing the specificity of antibodies against SNAP25 in sections of rat glabrous skin following treatment with either onabotulinumtoxinA (30 U/kg) or vehicle. (**A**–**E**) Confocal images of rat skin injected with onabotulinumtoxinA and probed with (**A**) commercial mAb (SMI-81R) against full-length (206) and cleaved (197) SNAP25; (**B**) a second commercial mAb (MC-6050) against SNAP25_197_ and SNAP25_206_; (**C**) commercial mAb (MC-6053) against SNAP25_197_; (**D**) Ab632-rMAb against SNAP25_197_ and (**E**) RGT-1092 pAb against SNAP25_197_; (**F**–**J**) Confocal images of control rat skin injected with vehicle and probed with the same five antibodies. SNAP25-IR signal is in green. Arrows (**F**–**J**) point to IR-signal within nerve fibers from vehicle treated rat skin. Asterisks ((**B**,**C**,**G**) and (**H**)) point to non-specific IR-signal within the lumen of blood vessels. BV, blood vessel; CT, connective tissue; Scale bar = 50 μm.

In rat glabrous skin, the SMI-81R antibody exhibited IR-signal in nerve fibers surrounding blood vessels (among other skin regions). As expected for this antibody, the SNAP25-IR pattern was identical in both onabotulinumtoxinA and saline-treated skin (Figure 3A,F). The MC-6050 monoclonal antibody demonstrated IR-signal primarily in nerve fibers from toxin-treated skin. However, slight IR-signal was also evident in nerve fibers from saline-treated skin (Figure 3B,G). The MC-6050 antibody also showed strong IR-signal in the lumen of blood vessels (Figure 3B,G). But as this particular IR-signal was never demonstrated by other SNAP25 antibodies, it was determined to be non-specific. Similarly, the MC-6053 monoclonal antibody showed IR-specific signal in nerve fibers surrounding blood vessels from both toxin and saline-treated skin, as well as non-specific signal in the lumen of blood vessels (Figure 3C,H). Once again, RGT-1092 pAb generated against SNAP25_197_ demonstrated IR-signal in both toxin-treated and saline-treated rat skin (Figure 3E,J), suggesting that despite its specificity in WB analysis, this antibody is not amenable for IHC. In clear contrast, Ab632-rMAb exhibited IR-signal in nerve fibers only in toxin-treated, but not in saline-treated skin (Figure 3D,I) supporting its superb specificity. Ab635-rMAb showed similar specificity as Ab632-rMAb to SNAP25_197_ in rat glabrous skin (Figure S3B,D).

Our samples of rat glabrous skin often contain underlying skeletal muscle, providing an excellent opportunity to validate our rMAbs on their ability to recognize BoNT/A-cleaved SNAP25 within motor nerve terminals (MNT). Similar results for antibody specificity were observed in MNTs as in other skin nerve fiber-types (Figure S4). While the SMI-81R mAb recognized both full length and BoNT/A-cleaved forms of SNAP25 in MNTs and axons, the commercial mAbs, MC-6050 and MC-6053 demonstrated IR-signal primarily in nerve fibers from toxin-treated skin. However, non-specific IR-signal was also observed in saline-treated tissue for these commercial mAbs (Figure S4F,G). In contrast, Ab632-rMAb exhibited IR-signal in MNTs and axons only in toxin-treated, but not in saline-treated skin (Figure S4D,H).

**Figure 4:**
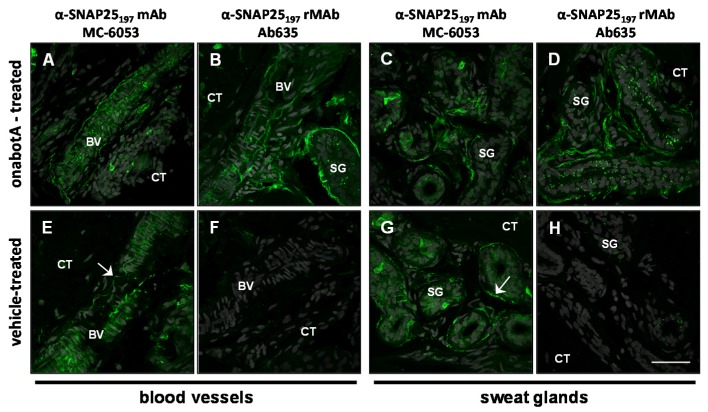
Immunohistochemical comparison of the commercially-available (MC-6053) mAb against SNAP25_197_ *vs.* Ab635-rMAb in sections of human back skin following treatment with either onabotulinumtoxinA (10U) or vehicle. Confocal images of blood vessels in onabotulinumtoxinA (**A**,**B**) and vehicle-treated (**E**,**F**) human skin probed with either (A,E) the MC-6053 mAb or (**B**,**F**) Ab635-rMAb. Sweat glands in onabotulinumtoxinA (**C**,**D**) and vehicle-treated (**G**,**H**) human skin probed with either (C,G) the MC-6053 mAb or (D,H) Ab635-rMAb. SNAP25-IR signal is in green. Arrows point to IR-signal within nerve fibers from vehicle treated human skin. BV, blood vessel; CT, connective tissue; SG, sweat gland; Scale bar = 50 μm.

Among the commercially available antibodies against SNAP25, the MC-6053 monoclonal antibody targeting SNAP25_197_ is most similar to our murine Ab635-rMAb with regard to the type and species of antibody (Table 1). We therefore performed a head-to-head comparison of the SNAP25-IR expression patterns between our Ab635-rMAb and the MC-6053 mAb in biopsy samples of onabotulinumtoxinA and saline-treated human back skin. The presumption was that since this was a probe of human tissue using a mouse antibody, the non-specific IR-signal (regardless of the source) would be minimal. In general, the IR-signal for both antibodies was observed in nerve fibers surrounding blood vessels and sweat glands within the skin (Figure 4). IR-signal for the MC-6053 mAb was observed in nerve fibers from both onabotulinumtoxinA and saline-treated back skin. In sharp contrast, IR-signal for our murine Ab635-rMAb was only observed in nerve fibers from onabotulinumtoxinA-treated, but not saline-treated human back skin (Figure 4) demonstrating its superior specificity and more importantly, its utility as a clinical diagnostics tool.

### 2.3. Immunocytochemistry

**Figure 5:**
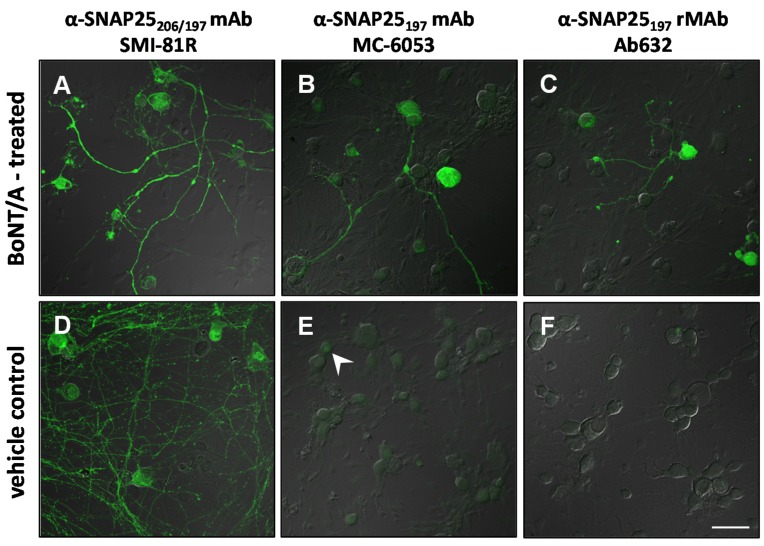
Confocal images of BoNT/A (3 nM)- or vehicle-treated dorsal root ganglia (DRG) cultures probed with antibodies to different forms of SNAP25. (**A**–**C**) DRG cultures exposed to BoNT/A for 3 hr and later stained with (**A**) commercial (SMI-81R) mAb against full-length (206) and cleaved (197) SNAP25, (**B**) commercial (MC-6053) mAb against SNAP25_197_ and (**C**) Ab632-rMAb against SNAP25_197_; (**D**–**F**) Control DRG cultures exposed to vehicle and probed with the same three antibodies. SNAP25-IR signal is in green. Arrowhead in (**E**) points to a neuronal soma exhibiting background labeling. Scale bar = 50 µm (**A**–**C**,**E**,**F**), 34 µm (**D**).

Indeed, some antibodies may work better for one assay/indication over another. Therefore, in order to complete our analysis, we compared the IR-signal from several of the antibodies in DRG cell cultures that were treated with either BoNT/A (3 nM) or saline. As in the tissues, the SMI-81R antibody showed strong IR-signal in both BoNT/A and saline-treated cultures (Figure 5A,D). Both the MC-6053 commercially available mAb and our human Ab632-rMAb demonstrated specific SNAP25_197_-IR signal in neuronal cells from BoNT/A-treated cultures (Figure 5B,C). No signal was detected in saline-treated cultures (Figure 5E,F). However, the MC-6053 mAb exhibited a faint background signal over the neuronal soma in the saline-treated cultures (Figure 5E). The results listing each antibody and its apparent specificity to either full length (206) or BoNT/A-cleaved (197) forms of SNAP25 in individual assays from the current study are summarized and presented in Table 2.

**Table 2:** Summarized results of antibody specificity to either full length (**206**) or BoNT/A-cleaved (**197**) forms of SNAP25 in individual assays.

Antibody	Specificity	Western blot	Rat bladder	Rat skin	Human skin	Rat DRG culture
SMI-81R	SNAP25_206/197_	206 + 197	206 + 197	206 + 197	n/t	206 + 197
MC-6050	SNAP25_206/197_	197	197	206 + 197 + b	n/t	n/t
MC-6053	SNAP25_197_	197	197	206 + 197 + b	206 + 197	197 + b
Ab632	SNAP25_197_	197	197	197	n/t	197
Ab635	SNAP25_197_	197	197	197	197	n/t
RGT-1092	SNAP25_197_	197	206 + 197	206 + 197	n/t	n/t

n/t = not tested; b = background.

## 3. Discussion

The presence of active BoNT/A in cells expressing SNAP25 can often be determined by using a selective antibody against the cleaved substrate (SNAP25_197_). In the present study, we introduce several rMAbs that were developed in-house against SNAP25_197_ and compared their immuno-reactive signal against that of commercial antibodies using a variety of different methods (Table 2). Both our human and murine rMAbs consistently detected SNAP25_197_ in all assays and on different tissues, and as expected, did not detect full-length SNAP25 (SNAP25_206_). This was not the case with other purportedly SNAP25_197_-selective antibodies, which displayed variable assay-dependent specificity. These results confirm that the BoNT/A-cleaved SNAP25 epitope is difficult to target specifically with an antibody without also recognizing the intact SNAP25 protein, which could lead to potential misinterpretation of results if the proper controls are not in place. Therefore, any given SNAP25_197_ antibody should be tested under multiple conditions and tissue types to ensure its fidelity in detecting the presence of BoNT/A-cleaved SNAP25.

### 3.1. Anti-SNAP25_197_ Polyclonal Antibodies

Previous studies in the literature characterizing the activity of BoNT/A in the rodent CNS have relied, in part, on a proprietary rabbit pAb against SNAP25_197_. This pAb was employed in WB and IHC assays to track SNAP25_197_ protein expression in the rat hippocampus, retinotectal pathway and in facial motor neurons following BoNT/A injections into those regions [17,20,21]. In addition, other investigators utilizing this antibody have shown SNAP25_197_-immunoreactive nerve fibers within dorsal and ventral spinal cord following BoNT/A peripheral and intraneural injections [18,19]. In all, the SNAP25_197_-positive staining observed in these studies was purported as being indicative for the presence of active BoNT/A in both primary and second-order neurons. Consequently, these and other data have formed the basis for an emerging hypothesis that BoNT/A administered peripherally undergoes cellular transport and transcytosis within the CNS [30,31].

Curiously, in a recent study focusing on a chronic constriction injury mouse model of neuropathic pain, investigators using this same rabbit anti-SNAP25_197_ pAb reported IR-staining primarily in GFAP-labeled Schwann cells in the sciatic nerve and in spinal astrocytes following interplantar injection of BoNT/A [32]. In contrast, a separate study using a rat orofacial pain model examined SNAP25_197_ expression in the trigeminal ganglion following BoNT/A injections into the whisker pad and found no IR-staining in the surrounding glia or in second-order neurons with the same pAb [33]. While the observed differences between these various studies may be due to the experimental setup, animal species used or sensory region examined, these discrepancies and the lack of critical control experiments suggest that the IR-signal from the published anti-SNAP25_197_ rabbit pAb may be inconsistent and misleading.

The data from our own anti-SNAP25_197_ rabbit pAb support this view. Our rabbit pAb demonstrated clear selectivity for SNAP25_197_ in WB analysis as well as other *in vitro* assays [34], although some faint non-specific bands were still observed (Figure 1F and Figure S1). However, this pAb did not work well for IHC, as evidence by the IR signal observed in different tissues with or without BoNT/A treatment (Table 2). In fact, the results from our rabbit pAb in tissues were visibly different than those of the mAbs, indicating the advantage of using monoclonal antibodies, in general, for identifying the SNAP25_197_ antigen.

### 3.2. Anti-SNAP25_197_ Monoclonal Antibodies

Monoclonal antibodies for the detection of SNAP25 are commercially available (http://www.rdabs.com/). According to the accompanying documentation, MC-6050 is specific for both intact (SNAP25_206_) and BoNT/A-cleaved SNAP25 (SNAP25_197_) [28], while MC-6053 only recognizes the BoNT/A-cleaved form of SNAP25 [29]. Several studies have utilized the SNAP25_197_-specific commercial mAb (MC-6053) by IHC analysis to examine the distribution of SNAP25_197_ in the guinea pig bladder following injections of BoNT/A into the detrusor muscle [22,23]. These investigators found SNAP25_197_-IR in both parasympathetic and sympathetic autonomic innervation as well as in sensory nerve fibers within guinea pig bladder and intramural ganglia. In these studies, the specificity of the MC-6053 mAb was not assessed by WB, or any other type of analysis.

Our assessment of the commercial anti-SNAP25 mAbs revealed a diverse set of results. In WB analysis, the MC-6050 anti-SNAP25_206/197_ mAb only recognized SNAP25_197_ in both cortical and SiMa cell lysates, while the MC-6053 anti-SNAP25_197_ mAb recognized SNAP25_197_ in cortical, but not SiMa cell lysates. In IHC analysis of rat tissues, both mAbs demonstrated SNAP25_197_-specific signal in nerve fibers from onabotulinumtoxinA-treated bladder, but these mAbs did not show clear specificity in rat skin. Similarly, the MC-6053 mAb was not specific for SNAP25_197_ in human skin tissue, demonstrating IR-signal in nerve fibers from both onabotulinumtoxinA- and saline-treated skin. These mixed results may be due to the fact that both antibodies were generated to a relatively long (15-mer) peptide antigen sequence. This could produce antibodies that may not have selectivity across different species [16,35]. Nevertheless, the results observed with the commercial antibodies and the discrepancies with the manufacturer’s description casts doubts as to their specificity and broad utility for IHC. In contrast, the data presented herein using our SNAP25_197_ rMAbs demonstrate their excellent specificity, versatility and consistency for detecting the presence of active BoNT/A in a broad range of assays.

### 3.3. Conclusion

Site-specific antibodies are increasingly being used for both *in vitro* and *in vivo* analysis. These antibodies can detect sites of phosphorylation or sites of enzymatic cleavage and are invaluable tools for our understanding of the maturation, activity and degradation of proteins [16,35,36]. Within the field of botulinum neurotoxins, cleavage site-specific antibodies can help detect the activity of minute quantities of BoNT light-chain that may otherwise be very difficult to perceive. To that end, the use of polyclonal antibodies may have more limited utility because a mixed immunoglobulin population could be produced, not all of which would have the required specificity for the cleavage epitope [16]. Furthermore, the peptide antigen should be relatively short in order to reduce the possibility of generating antibodies that bind to a part of the sequence remote from the target epitope.

The anti-SNAP25_197_ mAb presented in the current study was initially screened and selected for its superior performance in IHC assays [26]. The rMAbs (Ab632 and Ab635) that were subsequently generated from this antibody demonstrated superb specificity to BoNT/A-cleaved SNAP25 in several different assays, including IHC. Furthermore, these rMAbs showed superior SNAP25_197_ specificity compared to other antibodies tested (Table 2). Accordingly, our rMAbs represent effective new tools for the detection of BoNT/A activity within cells and in clinical samples, and can be utilized in future studies to characterize the effects of BoNT/A in tissues of interest.

## 4. Materials and Methods

### 4.1. Antibodies

The following primary antibodies were used in this study (Table 1): mouse anti-SNAP25_206/197_ (SMI-81R) (Covance, Princeton, NJ); mouse anti-SNAP25_206/197_ (MC-6050) and mouse anti-SNAP25_197_ (MC-6053) (R&D Antibodies, Las Vegas, NV, USA); human recombinant anti-SNAP25_197_ (Ab632), mouse recombinant anti-SNAP25_197_ (Ab635) and rabbit anti-SNAP25_197_ (RGT-1092) (Allergan, Irvine, CA, USA); mouse anti-GAPDH (ab8245) (Abcam, Cambridge, MA, USA); rabbit anti-protein gene product 9.5 (PGP9.5; AbD Serotec, Raleigh, NC, USA).

### 4.2. Cell cultures

Rat cortical neurons were harvested from embryonic pups (E18) and digested in a papain dissociation system (Worthington Biochemical Corp., Lakewood, NJ, USA) at 37 °C for 15 min to obtain individual cells. Cortical cells were then transferred to Neurobasal medium containing B-27 supplements, 0.5 mM L-glutamine and penicillin/streptomycin (Life Technologies, Carlsbad, CA, USA). Rat dorsal root ganglia (DRG) harvested from neonatal pups (P7–P14) were pooled and digested in papain-containing HBSS (final concentration of 20 units of papain per mL in 1 mM L-cysteine) at 37 °C for 15 min. Ganglia were washed and subsequently digested in Ca^2+^/Mg^2+^-free HBSS containing Type 1 collagenase (1.7 mg/mL, Sigma, St. Louis, MO, USA) and incubated at 37 °C for an additional 15 min. The ganglia were then washed in Neurobasal-A media containing B-27 supplements, 0.5 mM l-glutamine, penicillin/streptomycin and 20 ng/mL 2.5S nerve growth factor (Life Technologies, Carlsbad, CA, USA) and gently triturated through Pasteur pipettes. Cortical and DRG cells were homogenously dispersed, plated onto poly-d-lysine/laminin-coated 12-mm coverslips (BD Biosciences, San Jose, CA, USA) placed in 100-mm culture dishes and grown for 6 to 7-DIV prior to treatment. All animal protocols and procedures were approved by the Allergan Institutional Animal Care and Use Committee and performed in accordance with NIH guidelines.

On select days, cultures (*n* = 2–9, depending on the antibody) were treated with or without 3 nM BoNT/A (150 kDa; Metabiologics, Madison, WI, USA) for 3 h at 37 °C. Following treatment, cells were rinsed with and then incubated in fresh culture medium overnight. Cortical cells were then washed with PBS, lysed in freshly prepared Lysis Buffer (20 mM Tris pH 7.5, 0.15 M sodium chloride, 1 nM EDTA, 1 mM EGTA, 10% Triton X-100 and one tablet of EDTA-free protease inhibitors) for 20 min on ice and then centrifuged at 4000 rpm for 20 min to eliminate debris prior to Western blot (WB) analysis. DRG cells were washed, fixed with 4% paraformaldehyde (EM Sciences, Hatfield, PA, USA) for 10–15 min and processed for immunocytochemistry according to the protocol below.

SiMa cells (DSMZ, Braunschweig, Germany) were cultured in BD Biosciences brand Collagen IV flasks (VWR, Radnor, PA, USA) with vented caps [24]. Growth media consisted of RPMI 1640, 0.1 mM Non-Essential Amino-Acids, 10 mM HEPES, 1 mM Sodium Pyruvate, 100 U/mL Penicillin, 100 µg/mL Streptomycin, and 10% Fetal Bovine Serum (Life Technologies, Carlsbad, CA, USA). Cells were treated with or without BoNT/A (0.01 nM) for 24 h at 37 °C. Following treatment, SiMa cells were washed with PBS, lysed in freshly prepared Lysis Buffer for 20 min on ice and then centrifuged at 4000 rpm for 20 min to eliminate debris prior to WB analysis.

### 4.3. Western Blot

For Western blot assays (*n* = 1–5 blots per lysate, depending on the antibody), we employed rat embryonic cortical neurons as well as a human neuroblastoma cell line (SiMa), which is known for its sensitivity to BoNT/A-mediated SNAP25 cleavage [24,25]. Total cell lysates from these cultures were separated by electrophoresis (Biorad TGX Any Kd or 4%–20% gels) and the gel was transferred onto a PVDF membrane (Bio-Rad, Hercules, CA, USA). Blots were blocked in buffer (5% dry milk in 1X TBS-0.1% Tween-20) for 1 h at room temperature and then incubated overnight at 4 °C with primary antibodies in blocking buffer. Following washes, blots were incubated with HRP-conjugated secondary antibodies (Bio Rad, Hercules, CA, USA) and developed by ECL Plus (GE Healthcare, Pittsburgh, PA, USA). A separate control blot was probed for glyceraldehyde 3-phosphate dehydrogenase (GAPDH) to show equal loading of cell lysate samples. In separate WB control studies, cell lysates from either BoNT/C- or BoNT/E-treated (Metabiologics, Madison, WI, USA) SiMa cells were prepared and analyzed in a similar manner.

### 4.4. Animals and Skin Biopsy Samples

Male Sprague Dawley rats (220–250 g; Charles River Laboratories, Wilmington, MA, USA) were used for this study (*n* = 2–18, depending on the antibody). Rats were pair-housed with free access to food and water on a 12 h light-dark cycle. All animal protocols and procedures were approved by the Allergan Institutional Animal Care and Use Committee and performed in accordance with NIH guidelines.

Human skin biopsy samples (1 mm dia.) from 4 patients injected with 10 U onabotulinumtoxinA (BOTOX^®^, Allergan, Irvine, CA, USA) and 2 patients injected with vehicle were obtained through a Phase 1 Allergan clinical study. The study was conducted in accordance with the Declaration of Helsinki, the guidelines and regulations for Good Clinical Practice and all relevant local and country privacy guidelines. The study protocol, informed consent, and all appropriate study-related documents were approved by the Institutional Review Board/Ethics Committee.

### 4.5. BoNT/A Preparation and Injection Procedures

Working solutions of onabotulinumtoxinA (BOTOX^®^, Allergan, Irvine, CA, USA) and BoNT/A (150 kDa; Metabiologics, Madison, WI, USA) were prepared in either 0.9% saline or 0.5% BSA/0.9% saline, respectively. For bladder injections, rats were first anesthetized and prepped for surgery. A lower midline abdominal incision was made, exposing the urinary bladder, seminal vesicles, and prostate gland. The urinary bladder wall was then injected at four equidistant sites along the midline circumference with 10 µL of onabotulinumtoxinA (2.5 U/kg) yielding a final toxin load of 10 U/kg. Control animals received 10 µL injections of vehicle (0.9% saline) into comparable target sites. For glabrous skin injections, onabotulinumtoxinA (30 U/kg) or vehicle was administered as a single intradermal (ID) injection (25 µL) into the center of the right hindlimb paw between the footpads. Adult human back skin was injected ID with either 10 U of onabotulinumtoxinA or vehicle.

### 4.6. Tissue Preparation

Rats were sacrificed at 2-days post-injection and bladders or the central portion of the planter surface of the hindpaws were harvested and fixed overnight at 4 °C in Zamboni’s fixative (American MasterTech, Lodi, CA, USA). Punch biopsy samples from human back skin were harvested 14-days post-treatment and fixed overnight in the same fixative. Tissues were washed and cryoprotected in 30% sucrose/PBS solution overnight at 4 °C. Bladder and skin samples were hemisected along the midline, embedded in Tissue-Tek O.C.T. (Sakura Finetek USA, Torrance, CA, USA) and stored frozen at −80 °C until sectioning. Tissue blocks were cryostat-sectioned (14 µm-thick), slide-mounted and slides were kept at −20 °C until use.

### 4.7. Immunocyto/Histochemistry

Slide-mounted tissue sections and cell culture coverslips were first blocked for non-specific signal in blocking buffer (1X PBS + 0.1% Triton X-100 + 10% Normal Donkey Serum) and then incubated with primary antibodies at the desired concentrations in blocking buffer overnight at 4 °C. Following several washes, sections and coverslips were incubated with secondary antibodies (Jackson ImmunoResearch, West Grove, PA, USA) diluted in blocking buffer for 2 h at 4 °C and then washed again. Coverslips with cultures were inverted and mounted onto microscope slides using Fluoromount-G (EM Sciences, Hatfield, PA, USA) containing 1.5 µg/mL DAPI (Roche Diagnostics, Indianapolis, IN, USA). Slide-mounted sections were coversliped using the same mounting media. Adjacent sections processed without primary antibodies served as negative controls to show background signal. Alternate sections were stained with Hematoxylin and Eosin (Thermo Fisher Scientific, Waltham, MA, USA) for better anatomical identification.

### 4.8. Data Analysis

For qualitative IHC analysis of rat tissues, a minimum of 3 sections per tissue per animal were analyzed in their entirety for SNAP25_197_-IR signal for each antibody. For human tissue analysis, whole biopsy samples (*n* = 50–60 sections per sample) were analyzed for SNAP25_197_-IR signal for each antibody. For qualitative immunocytochemical analysis, 3 coverslips per culture were analyzed for SNAP25_197_-IR signal for each antibody. Representative images from the analyzed tissues and cultures were captured using either a Zeiss LSM-710 confocal microscope (Carl Zeiss, Thornwood, NY, USA) with Zeiss ZEN software (Version 5.5, Carl Zeiss, Thornwood, NY, USA, 2009) or an Olympus FV1000 confocal microscope (Olympus, Center Valley, PA, USA) with Olympus Fluoview FV-10 ASW software (Version 4.1, Olympus, Center Valley, PA, USA, 2003–2013). Nerve fibers were identified on the basis of their morphology and neurochemistry using the pan-neuronal marker PGP9.5 as a counterstain. Western blots were scanned using a variable mode GE Typhoon 9410 imager (GE Healthcare, Pittsburgh, PA, USA) and analyzed with Typhoon Scanner Control software (Version 5.0, GE Healthcare, Pittsburgh, PA, USA, 2004) and ImageQuant Tools software (Version 3.0, GE Healthcare, Pittsburgh, PA, USA, 2002).

## Supplementary Materials

Supplementary materials can be accessed at: http://www.mdpi.com/2072-6651/7/7/2354/s1.
