# Supplementary material for: A Highly Specific Monoclonal Antibody for Botulinum Neurotoxin Type A-Cleaved SNAP25
Source: Toxins (Basel). 2015 Jun 24;7(7):2354–70. doi: 10.3390/toxins7072354 (PMC4516917; doi:10.3390/toxins7072354)
Supplement: Supplementary file 1 [file toxins-07-02354-s001.pdf]

Supplementary Materials

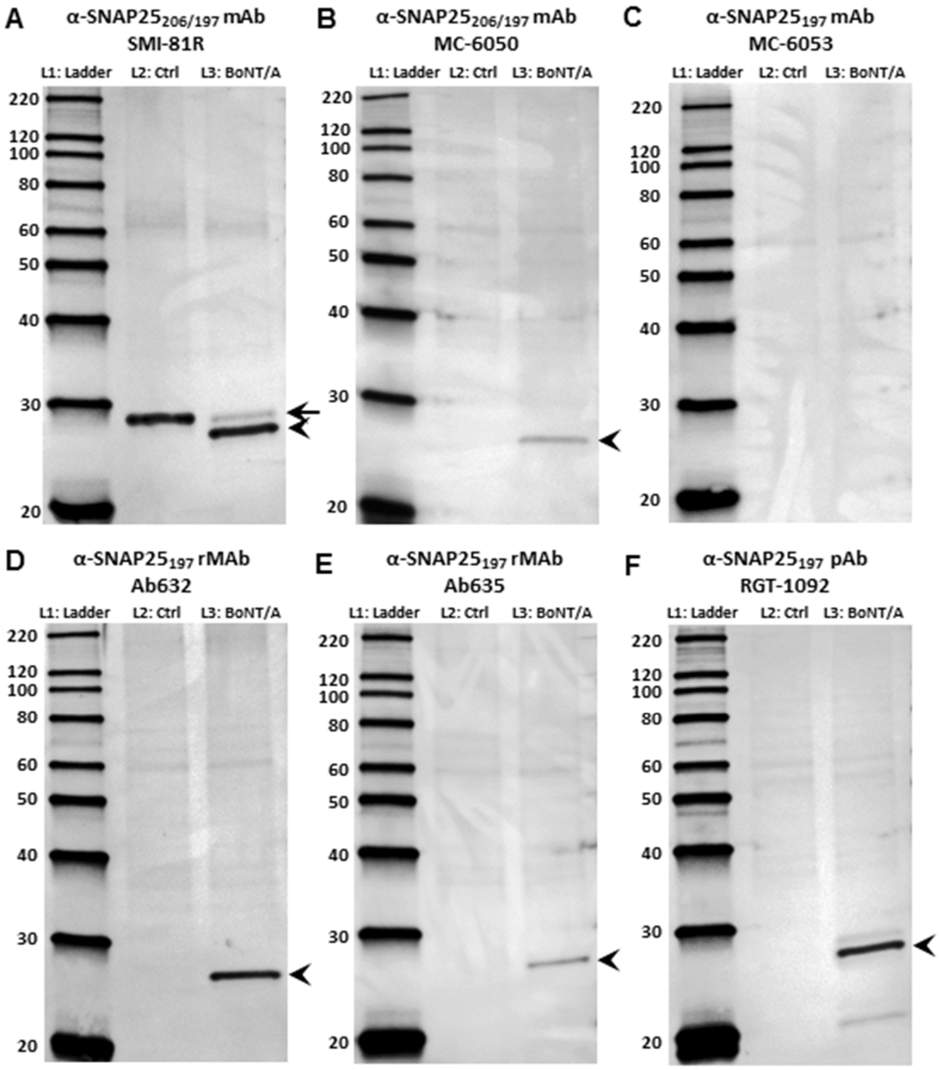

Figure S1. Western Blot analysis comparing the specificity of antibodies against SNAP25 using SiMa cell lysates treated with (L_3) or without (L_2) BoNT/A. (**A**) Blot probed with a commercially available anti-SNAP25 mAb (SMI-81R) that recognizes both the full-length (206) and cleaved (197) forms of SNAP25. In lane 2, only $SNAP25_{206}$ is detected, whereas in lane 3, both $SNAP25_{206}$ (arrow) and $SNAP25_{197}$ (arrowhead) are detected; (**B**) Blot probed with a commercially available anti-SNAP25 mAb (MC-6050) that reportedly recognizes both $SNAP25_{206}$ and $SNAP25_{197}$. Only $SNAP25_{197}$ appears as a single band in lane 3 (arrowhead); (**C**) Blot probed with a commercially available anti-SNAP25 mAb (MC-6053) that is reportedly specific for $SNAP25_{197}$. The antibody does not appear to recognize any bands; (**D**) Blot probed with Ab632 anti-$SNAP25_{197}$ rMAb. In lane 2, no band is detected, whereas in lane 3, a single band for $SNAP25_{197}$ is detected (arrowhead); (**E**) Blot probed with Ab635 anti-$SNAP25_{197}$ rMAb. In lane 2, no band is detected, whereas in lane 3, a single band for $SNAP25_{197}$ is detected (arrowhead); (**F**) Blot probed with RGT-1092 anti-$SNAP25_{197}$ pAb. This antibody primarily recognizes $SNAP25_{197}$ in lane 3 (arrowhead), although two faint bands are visible just above and below the $SNAP25_{197}$ band. Lane 1, protein ladder; Lane 2, untreated SiMa cell lysate; Lane 3, BoNT/A-treated (0.01 nM) SiMa cell lysate.

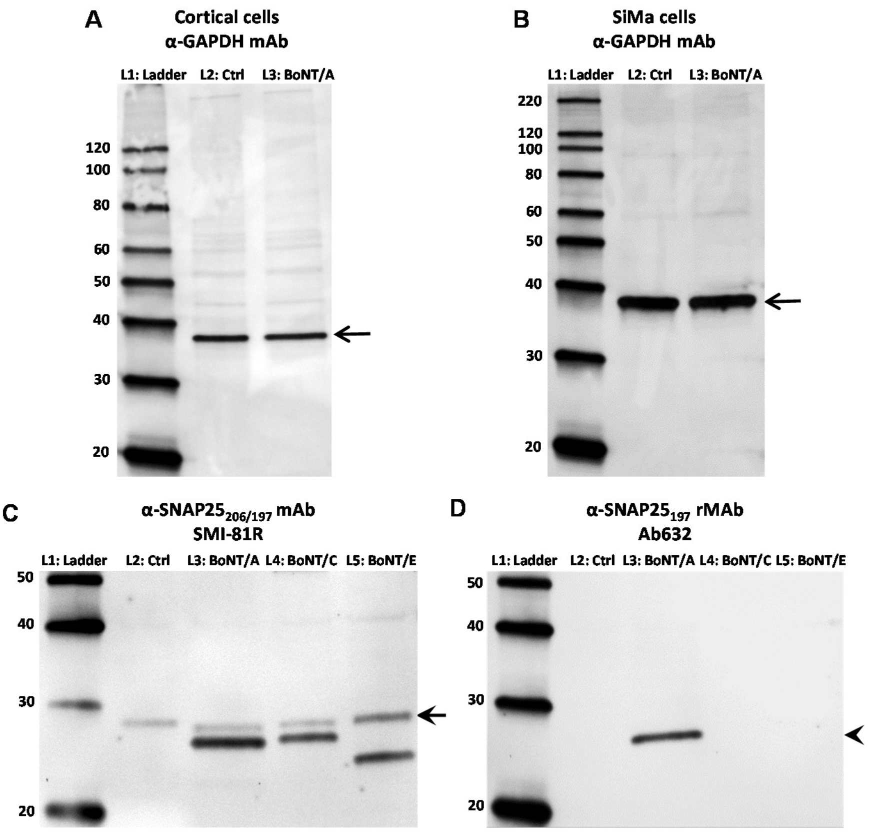

Figure S2. Control blots for the cortical cell studies in Figure 1 (**A**) and SiMa cell studies in Figure S1 (**B**) probed with anti-GAPDH mAb showing equal loading of samples in lanes 2 and 3. Lane 1, protein ladder; Lane 2, untreated cell lysate; Lane 3, BoNT/A-treated (3 nM) cortical cell lysate or (0.01 nM) SiMa cell lysate; (**C**,**D**) Western Blot analysis showing the epitope specificity of Ab632-rMAb using SiMa cell lysates treated with BoNT/A (L_3), BoNT/C (L_4), BoNT/E (L_5) or with no toxin (L_2). (**C**) Blot probed with a commercially available anti-SNAP25 mAb (SMI-81R) that recognizes both the full-length (206) and cleaved (197 for BoNT/A, 198 for BoNT/C and 180 for BoNT/E) forms of SNAP25. In lane 2, only $SNAP25_{206}$ is detected, whereas in lane 3, both $SNAP25_{206}$ (arrow) and $SNAP25_{197}$ are detected. In lane 4, both $SNAP25_{206}$ (arrow) and $SNAP25_{198}$ are detected and in lane 5, both $SNAP25_{206}$ (arrow) and $SNAP25_{180}$ are detected; (**D**) Blot probed with Ab632 anti-$SNAP25_{197}$ rMAb. In lane 2, 4 and 5, no band is detected, whereas in lane 3, a single band for $SNAP25_{197}$ is detected. Lane 1, protein ladder; Lane 2, untreated SiMa cell lysate; Lane 3, BoNT/A-treated SiMa cell lysate; Lane 4, BoNT/C-treated SiMa cell lysate; Lane 5, BoNT/E-treated SiMa cell lysate.

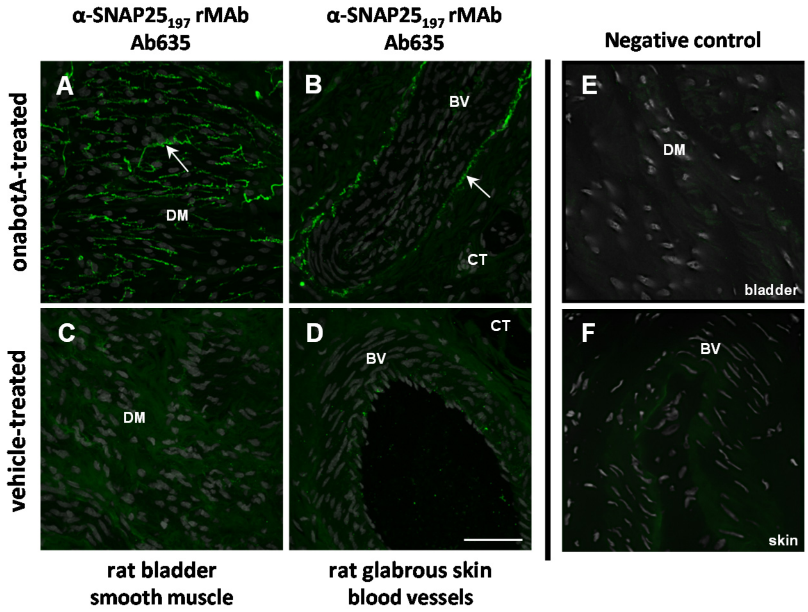

Figure S3. Immunohistochemical analysis showing the specificity of Ab635-rMAb in sections of rat bladder and glabrous skin following treatment with either onabotulinumtoxinA (10 U/kg, bladder; 30 U/kg, skin) or vehicle. (**A**,**B**) Confocal images of rat bladder detrusor muscle (**A**) and blood vessels within rat glabrous skin (**B**) showing IR-signal (arrows, green) in the nerve fibers following onabotulinumtoxinA treatment; (**C**,**D**) Confocal images of control rat bladder and skin injected with vehicle; (**E**,**F**) Confocal images of adjacent sections of rat bladder (**E**) and glabrous skin (**F**) processed without primary antibodies showing only background staining. DM, detrusor muscle; BV, blood vessels; Scale bar = 50 µm.

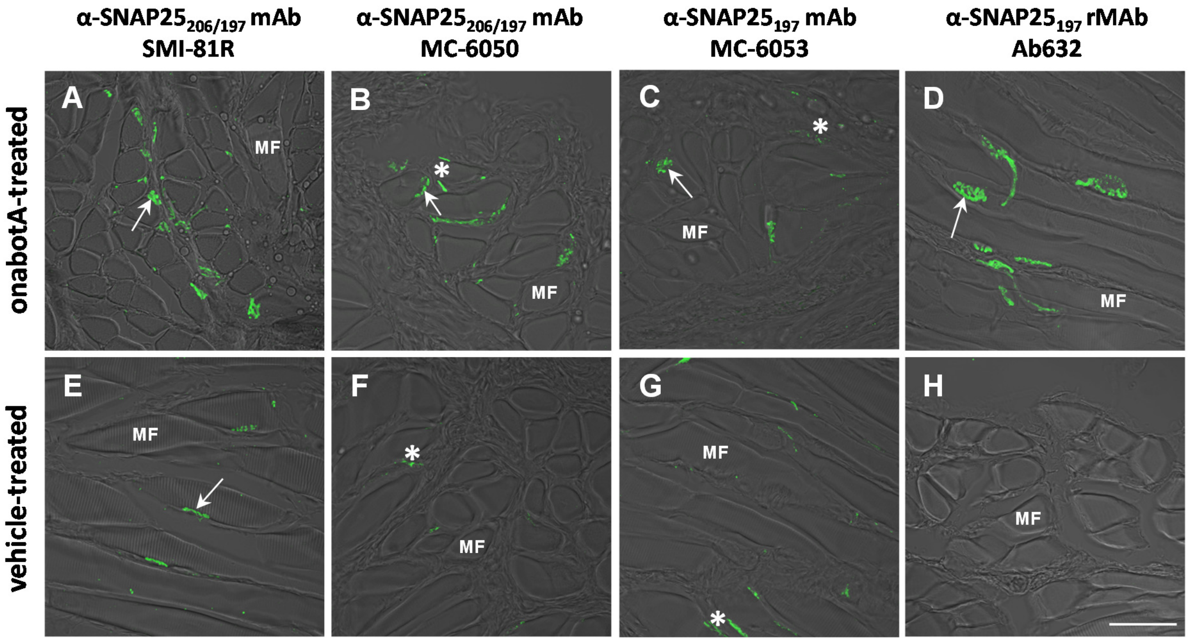

Figure S4. Immunohistochemical analysis comparing the specificity of antibodies against SNAP25 in skeletal muscle underlying rat glabrous skin following treatment with either onabotulinumtoxinA (30 U/kg) or vehicle. (**A**–**D**) Confocal images showing motor nerve terminals (MNT, arrows) within the underlying muscle of the rat paw injected with onabotulinumtoxinA and probed with (**A**) commercial mAb (SMI-81R) against full-length (206) and cleaved (197) SNAP25; (**B**) a second commercial mAb (MC-6050) against $SNAP25_{197}$ & $SNAP25_{206}$; (**C**) a commercial mAb (MC-6053) against $SNAP25_{197}$ and (**D**) Ab632-rMAb against $SNAP25_{197}$; (**E**–**H**) Confocal images from control rat paw injected with vehicle and probed with the same four antibodies. SNAP25-IR signal is in green and DIC illumination was used to delineate the underlying muscle fibers (MF). Arrow (**E**) points to IR-signal within a MNT from vehicle treated rat paw; asterisks (**B**,**C**,**F** and **G**) point to non-specific IR-signal within the muscle. Scale bar = 50 μm.
